# Items and Miscellany

**Published:** 1890-06

**Authors:** 


### ITEMS AND MISCELLANY

CORRECTION.—On page 165 of May issue of the JOURNAL, the title of Dr. Key's paper appeared as "Report of Seven Cases of Ovariotomy in the Last Ten Years," while it should have been "Laparotomy.—Report of Seven Cases in Last Year."

Dr. L. H. Jones, of this city, has been elected to the chair of Chemistry in the Atlanta Medical College, left vacant by the resignation of Dr. Hunter P. Cooper. Dr. Jones has had much practical experience as a chemist, and is in every respect well qualified for the place.

Dr. J. Madison Taylor, in *University Medical Magazine*, May,

1890, says of Sulphonal:—"In a large experience in the use of sleep persuaders, I have never known sulphonal to exhibit those treacherous qualities with which it is accredited." As a motor depressant he recommends that five to ten grains be given twice or thrice daily. As a hypnotic he prefers to begin in the afternoon, and give ten grains at a dose every two hours, until the quantity necessary to produce sleep has been administered by bed time, beginning earlier or later as the case demands. In conclusion he says:—"My feeling is, however, that in sulphonal we have a weapon which very often is effective where others fail, and freer from objectionable properties than many."

---

FREUND (H. W.). "Codeia in diseases of women."—*Centralblatt für Gynäkologie*, No. 2, 1890.

FOLLOWING Lauder-Brunton's recommendation of codeia (in *British Medical Journal*, Jan. 9. 1888) for "pains in the bowels and lower part of the abdomen," Freund has made observations as to its usefulness in diseases of women. He finds that while in pain which proceeds from the uterus codeia gives temporary relief, in exudations of the pelvic peritoneum and connective tissues and affections of the tubes it is useless. On the other hand, in ovarian troubles Freund finds it to be of great value. He gives three times daily one pill containing half a grain of codeia. This dose promptly relieves all ovarian pain, whether of inflammatory origin or so-called simple neuralgia. The action of codeia is a purely local one. It does not stupify, above all things, nor does it interfere with the appetite or regular movement of the bowels.—*Medical Chronicle*, *May*, *1890*.

---

FALK. "Hydrastinin in uterine hemorrhage."—*Archiv für Gynäkologie*. Band 37, Heft 2, 1890.

FALK has studied the action of hydrastinin experimentally upon animals, and clinically in diseases of women. Experimentally he finds that it exercises a marked action on the peripheral bloodvessels, producing contraction of them, this action being a peripheral one on the vessels themselves. Clinically, he finds that it is a certain remedy against uterine hemorrhage. Especially good is its action in cases of hyperplastic endometritis, in congestive dysmenorrhœa, and in bleeding from the virgin uterus, the beneficial action being the result of a diminished blood-flow to the pelvic organs. In myomata also it checks hemorrhage. Less certain results are obtained in those forms of chronic metritis where the uterus is enlarged by a hyperplasia of the connective tissue, while the muscular tissue has lost its contractile power. Uncertain also is the result in bleeding of neurotic origin. Excellent results are obtained by using hydrastinin as a prophylactic against excessive menstruation. It acts more rapidly and certainly than the fluid extract of hydrastis canadensis, and is of use often in cases where ergotin produces no result. Falk uses it hypodermically, employing a 10 per cent. watery solution of the hydrochlorate of hydrastinin.—*Medical Chronicle, May, 1890.*

---

Collischonn. "Phenacetin as an anti-rheumatic."—*Deutsch med. Woch.*, 1890, No. 5. Abstract in *Therap. Monatsh.*, 1890, p. 138.

Since using larger doses of phenacetin, Dr. Collischonn has had most gratifying results in the treatment of rheumatism, and has given up the use of salicylic acid on account of its common unpleasant after effects. Dr. Collischonn himself took some 120 grms. (3½ oz.) of salol in the course of three weeks without any effect, while two doses of 2 grms. (30 grs.) of phenacetin taken in the afternoon led to marked relief within two days. Phenacetin was in all the cases, with one exception in which there was persistent vomiting, very well borne by the digestive organs, and save somewhat severe sweating, which occurred in one case after the administration of four doses of 1 grm. (15 grs.) Dr. Collischonn has not observed any ill-effect. The fever disappeared at once; the temperature, indeed, was often subnormal, as also was the heart-rate, the pulse being some 10—15 beats below the average.

In no case was any rash observed. For purely practical purposes Dr. Collischonn divides cases of rheumatism into four classes, and in these the therapeutic effect is different, as will be seen by the subjoined table of his cases:—

I. Polyarthritis rheumat. acut. afebril.: 29 cases, of which 19 cases were cured.

II. Polyarthritis rheumat. acut. afebril.: 19 cases, of which 12 cured.

III. Rheumat. muscul. afebril.: (very slight cases not included) 12 cases, of which 8 cured.

IV. Rheumat. musculo-artic. afebril.: 10 cases, of which 2 were cured.

Acute articular rheumatism responded most quickly to the drug; the afebrile forms respond much less to phenacetin. The author recommends at least a trial of phenacetin in all cases of rheumatism, best administered in four doses of 1 grm. (15 grs.), or two of 2 grms. (30 grs.)—*Medical Chronicle*, *April*, *1890*.

---

"Phenacetin in whooping-cough."—*Apotheker Zeitung*, Jan. 19, 1890.

DR. KATZ records, *Verein*, *f. inn. Med.*, that he has found phenacetin of great use in three cases of pertussis. Dr. Michaelis has also found it of great use as a sedative, in doses of 4 to 10 grains in children suffering from the same disease, in two cases in which the cough was of a fortnight's duration. Dr. Michaelis observed great relief after the administration of 4 grs. to the younger child (1½ years), 10 grs. to the elder (4 years).

Dr. Sleimann records a case (*Mun. med. Wschr.* 12, 1890) in which, after using antipyrin with no benefit, he had a most satisfactory result with phenacetin; the attacks became much reduced during the day, but occurred again at night, when the drug was not administered; in none of the cases did he observe any evil effects.

Dr. Irwin reports a case (*Deutsche med. Zeit.*, Dec. 15, 1889) in which he had a surprising effect; after using, without avail, belladonna, quinine, antipyrin, and other remedies, he administered phenacetin, which was followed by an immediate alleviation of the severe symptoms; given in doses of 0.03 grms. (0.5 gr.) every four hours, the attacks were at once cut short, and in the first night the child slept 6½ hours ; the drug was administered in glycerine, in which phenacetin is soluble.—*Medical Chronicle, April, 1890.*

---

FRANZ (Breslau). "On the action of sulphonal."—*Therapeutische Monatshefte*, No. 3, 1890, p. 119.

THIS is a communication on the results obtained with "Sulphonal Bayer" in surgical practice. Dr. Franz records his experience of 82 patients to whom, in all, 260 doses were administered. In no single case did he observe the slightest ill-effect follow the use of the drug. In nervous sleeplessness, in all 26 cases, with 82 single doses, sulphonal failed only four times in one and the same patient—a very anæmic man, suffering from phthisis pulmonalis, who was operated on several times for tubercular abscesses ; patient was too accustomed to morphia. In all the other cases the patients slept well after sulphonal, the sleep being natural and refreshing ; in no cases was there complaint of giddiness, heaviness, or headache, nor was there any ataxic symptom. In the one case in which the sleep was not satisfactory, the patient complained once after a dose of 3 grm. (45 grs.) of hallucinations; otherwise he made no complaint. In one case he succeeded in breaking the morphia habit by the use of sulphonal. In the greater number of cases the maximum dose was 1 grm. (15 grs.), but in the case of patients suffering pain from wounds or other causes, the dose must be larger, 2 grm. (30 grs.) *pro dosi* was enough to cause sound sleep. In one case a severe supra-orbital neuralgia disappeared after the sleep following a dose of 1 grm. (15 grs.). Immediately after chloroform narcosis, sulphonal was very well borne, the usual headache and vomiting being rather diminished than increased ; the sleep obtained in these cases immediately after operation was in some cases light, and easily broken, but refreshing. Several patients slept better after the administration of sulphonal than after morphia, which was given to them alternately. With children the results were especially satisfactory ; in 17 cases, doses varying from 0.5 to 1 grm (7½ to 15 grs.) were given, and a deep, refreshing sleep followed, absolutely free from any unpleasant after-effect. The drug was administered in the form of tablets, and somewhat early (6 to 8 p. m. in most cases of agrypnia nervosa), sleep ensued in about an hour ; where, however, patients suffered from pain, the sleep was somewhat longer delayed ; possibly the somewhat insoluble form in which the drug was administered had some influence here. Dr. Franz has observed no ill-effect follow the long-continued use of the drug, and this is well shown in the case reported by Dr. Steiner (*Therap. Monatsh.*, 1889, p. 460).—*Medical Chronicle, April, 1890.*

---

W. S. Christopher presents the following summary in concluding a paper on Summer Complaint:

1. Various forms of abnornal fermentations occur in the bowels, and when they occur in infants, and produce symptoms, they constitute the immediate cause of the collection of diseases known as summer complaint.

2. Summer complaint so defined includes putrefactive constipation and all forms of diarrhœa and dysentery not diphtheritic in origin nor symptomatic of septicæmia.

3. The three great predisposing causes of summer camplaint, viz., hot weather, overcrowding, and bottle-feeding, are to be regarded as acting solely as adjuvants to fermentation.

4. The diet during summer complaint should be determined entirely by the conditions within the bowels, and not by theoretical ideas as to Nature's food.

5. At least two well-marked forms of abnormal intestinal fermentation may be recognized clinically,—viz., the putrid and the acid.

6. In the putrid fermentation, carbohydrates should constitute the food, and in the acid form albumen should be the only food.

7. Milk containing, as it does, both proteids and carbohydrate, should be prohibited in all forms of intestinal fermentation. When properly sterilized, food can be given; nursing babies with severe summer complaint should be taken from the breast.

8. All food administered, of whatever type, should be aseptic.

9. In addition to regulating the diet on the foregoing principles, the treatment should include laxatives and intestinal antiseptics.

10. The lesions are to be regarded as the results of the fermentation, and are more marked in proportion to the duration of the disease.

11. The lesions assist in prolonging the disease, and in all probability act by providing a habitat for the micro-organisms, and by their secretions furnishing the micro-organisms with material with which to maintain their biological activity.

12. In chronic cases, where well-marked lesions may be supposed to exist, lavage of the large intestine and of the stomach, with appropriate antiseptics, is indicated.

13. Opium is contraindicated except in persistent acid fermentation, which threatens to produce anatomical lesions.—*Archives of Pediatrics, May, 1890.*

---

### CHLOROFORM VERSUS ETHER.

Surgeons cannot be content to leave the claims of these anæsthetics to physiologists or professional anæsthetists. Some of us have been for many years in the habit of superintending the administration of one or other of these drugs to hundreds of cases per annum. We may leave it to the anæsthetist to observe and report the minuter details of their action, but as to their action in the gross, or their fatality, we are quite competent to form our own views, and to speak for ourselves. The appeal is to clinical experience, and I trust apparent egotism will be pardoned. During a medical career of twelve years, dating from the time when I began attending the operations at the Edinburgh Royal Infirmary, I have never seen a case of death from chloroform.

During the last eight years at the Kashmir Mission Hospital, upwards of 11,000 operations have been performed. In over

3,000 cases chloroform has been administered in my presence; not a single fatal case has occurred. The cases in which serious danger has threatened might be counted on the fingers of one hand. None of these cases were due to any heart affection; it was a question of arrested respiration. Once the patency of the respiratory tract was secured, and a few artificial respiratory movements were performed, all danger passed away. Promptitude is necessary, but it is not a quality in which surgeons are deficient. It is to general surgeons not to anæsthetists that we are indebted for the knowledge how to meet the danger.

As far as the inhabitants of Central Asia and North India are concerned, chloroform may be regarded as a perfect anæsthetic. True, the beer drinking Tibetans occasionally struggle before succumbing to its influence, but of other races—Yarkandis, Hillmen, Pathans, Dards, Kashmiris, etc.—it may be said that to 99 per cent. chloroform may be given deeply and its administration prolonged without a drawback—no cardiac weakness, no bronchial irritation, very rarely signs of an over-dose. That no powerful anæsthetic is free from risk is a truism. My old teacher, Professor Chiene, used to say that it was like a fast train passing many stations without stopping, and halting or fluctuating between the signal point of semi-conscious reflexes and the terminus of cessation of vital reflexes, namely, respiratory stoppage, arrest of heart action, death. Let every driver beware the pace, watch the signals, and he will be safe. Such is the theory I learnt; such is my experience; others with wider experience say the same. Opposite theories cannot falsify extensive experience, even of individuals.—*Arthur Neve, F. R. C. S. Ed. The British Medical Journal, February 8th, 1890.*

---

### GASTRO-ENTEROSTOMY WITH SENN'S APPROXIMATION PLATES.

(Under the care of GEO. S. STANSFIELD, M. R. C. S.)

The extreme value of Professor Senn's bone plates and Mr. Jessett's experiments therewith, receiving apparently their first confirmation in the human subject in Mr. Clarke's case, recorded in the *Journal* of November 16th, 1889, warrants my submitting the following case to the profession:

W. G., aged 53, a laborer, applied for treatment on December 4th, 1889. He complained of pain, which was almost constant, but was always aggravated by any food, whether solid or fluid. He did not locate the pain in any particular small spot, but drew his hand across the epigastrium, saying "Just across here." He rarely vomited; latterly he had been afraid to eat, and as a result was extremely emaciated. If importance is attached to cachexia, he had certainly the cachexia of cancer. He had consulted many doctors, among whom there existed a considerable diversity of opinion as to his malady. He had been ill many months, and as his life was perfectly miserable, he was willing to submit to any operation that promised relief. Upon examination a tumor could be indistinctly felt a little above and to the right of the umbilicus.

On December 16th he was placed under chloroform, and an incision about three inches long made in the middle line downwards from the ensiform cartilage. Pushing aside the colon, a hard mass was found in the upper part of the right lumbar region, somewhat globular, and about four inches in diameter. The incision was lengthened by another inch, so that a better examination might be made. The third part of the duodenum was the portion implicated; the adhesions were so extensive as to preclude removal.

On the hypothesis that the pain was caused, or intensified, by the food having to force its way through an almost closed canal, it seemed to me that a communication between the stomach and jejunum would give a large amount of relief. Such a communication was therefore made exactly in the way described by Mr. Clarke, excepting that I had no occasion to use Lembert sutures, beyond one in the stomach, having accidentally made its incision a little too large. Deep and superficial sutures of chromicised cat-gut closed the parietal wound. There was no bleeding of any consequence, and no antiseptics were used.

The operation was completed in half an hour; towards the close the patient was very collapsed. Ether was given hypodermically, and he was not moved to his bed for two hours.

Beef-tea enemata, with liq. morphine, gtt. xx, were given every three hours. He vomited four times, and was greatly distressed by hiccough. At the end of twenty-four hours the morphine was stopped. His progress was uninterrupted.

On the fourth day after operation he was allowed beef-tea by the mouth. On the sixth day he had a little bread and milk, for which he had a great desire. On the tenth day the wound was examined for the first time, and was found completely healed by first intention. On the twenty-fourth day he got up. On the thirty-third day he was weighed, and found two pounds heavier than on the day of operation. The temperature never rose above the normal line, and the bone plates were never seen again. He is now free from pain, and eats food heartily.

It may be asked, why was not the first portion of the duodenum joined to the jejunum, instead of opening the stomach, seeing that the pylorus was perfect? Simply because the coil of jejunum used and the stomach came into proximity much more easily, and therefore avoided dragging, and in view of the patient's vomiting and hiccough in the first twenty-four hours, I attribute no small measure of his recovery to the freedom from dragging which existed.

I do not apprehend any trouble from the bile and pancreatic fluid, feeling confident that when the duodenum becomes completely closed, regurgitation into the stomach, and thence by the new passage, will not be troublesome.

I am indebted to Dr. L. McWhannell, senior house-surgeon, for careful notes from which the above is condensed.—*The British Medical Journal*, *February 8th*, *1890*.

---

Dr J. G. Johnson in a paper on "Disease Germs and disinfectants," published in May issue of *Brooklyn Medical Journal*, concluding his remarks on diphtheria makes the following summary:

"Diphtheria is produced by a disease-germ that invades the tonsil by preference. This germ secretes a poison similar in its effects on the human system to snake-poison. This poison destroys life by overwhelming the nervous system. That corrosive sublimate is a certain germicide, and should be applied locally ; kill the germ before it kills your patient. As the tendency is to death by exhaustion, the patient should be kept in bed from the first and freely stimulated.

During the whole time the patient should gargle frequently with strong lemonade, or one part of vinegar to four of water, as also should those attending on him.

DAMAGES FOR SLANDER.—At a recent decision in a suit for damages on account of slanderous language in the case of Cruikshank *vs.* Gordon, the plaintiff was awarded $1,600 damages. Defendant used the following language in talking to some of Dr. Cruikshank's patients : "The doctor had treated the child for malaria while it had another and entirely different disease ; he nearly killed the child and would have killed it if another doctor had not been called in."

"Among the legal questions involved in the case, there was one which will probably be appreciated by medical men fully as much as by those versed in the law. The plaintiff came into court without alleging, and probably without being able to prove, that he had sustained actual damage by reason of anything the defendant had said. Every one of his patients to whom the defendant had addressed defamatory words appeared in court to testify on the plaintiff's behalf ; and none were shown to have lost confidence in him as a physician. So far as related to that part of the language uttered by the defendant which denied the plaintiff's competency as a physician in general, such evidence of actual damage was clearly not required, the rule being well-settled that when a man is falsely said to be incompetent in his vocation, whatever that may be, the law presumes him to be necessarily damaged without proof ; the mechanic standing in this respect on the same footing with members of a learned profession. In some instances, as in the present case, it might be possible to show that this presumption was incorrect in fact ; but such proof would not be received."—*Brooklyn Medical Journal, May, 1890.*

DUHRSSEN. "The treatment of post-partum hemorrhage."—*Sammlung klin. Vorträge*, No. 347. Summary in *Centralblatt für Gynäkologie*, No. 10, 1890.

THE author strongly recommends a method of which he has had large experience—viz., tamponade of the uterus, and even of the whole genital tract, as the most reliable method of stopping hemorrhage in atony of the uterus after the completion of labor. It may also be employed to stop hemorrhage arising from laceration of the cervix. He regards it as the most quickly applicable, the least dangerous and most certain method. Dührssen refers to 79 cases in which the method of tamponade was resorted to by himself and others, and he gives an analysis of 65 cases. He shows that the objection that the gauze tampon would prevent the contraction of the uterus is erroneous, and that, on the contrary, all observers declare that it causes powerful contraction of the uterine musculature, and the uterus, instead of becoming flaccid again, continues in a state of retraction. The tamponade, then, acts by producing contraction as well as by permitting compression, and all parts of the uterus, including the placental site, are affected alike. In the cases that were fatal, in spite of the application of the tampon, the cause of the fatal result lay in delay. Everything else was tried first, hot and cold irrigation, ergotin, etc., and the patients were *in extremis* before the introduction of the plug. Even when the hemorrhage comes from a rent in the lower uterine segment, the tamponade is a suitable and effective method of treatment. If the source has been diagnosed as cervical, the tampon stops hemorrhage until appliances for suturing can be gotten ready; and if the site is not made out at first, it will be discovered afterwards under more favorable conditions, and the injury can then be dealt with. Tamponade is therefore the best method in all doubtful cases.

The material of which the tampon is made must be aseptic. Improvised materials, such as strips of linen, can be sterilized by boiling for five minutes in water. The best material to carry, with a view to emergencies, is absorbent gauze. The rough surface is an advantage, and such material does not readily part with its fluid.

Tamponade is not contraindicated in patients who have been already infected. It may be made the vehicle by which disinfectants are conveyed into the cavity of the uterus, and in this way the process of decomposition may be hindered, and the injured surfaces protected from infection. The danger of carrying septic matter from the vagina into the uterus can be reduced to the minimum, by thorough cleansing of the vagina as a preliminary. The extreme care to prevent infection, which every obstetric operation requires, must be bestowed by the medical attendant. The *technique* of the operation is very simple. Dührssen carries with him sterilized or iodoform gauze in a leaden box, and when the tampon has to be introduced, he thoroughly irrigates the vagina with hot water, and then drawing down the uterus with a vulsellum, he packs the gauze into the uterine cavity by means of suitable forceps, without ever touching the material with his fingers. The vagina is then packed with gauze or cotton wool. An anæsthetic is not necessary.

The author is so satisfied with the results that he already begins to recommend a "prophylactic tamponade"—that is, the introduction of the tampon in every case of considerable hemorrhage after the expulsion of the placenta. His remarks suggest the ready abuse of a proceeding which, within limits, may be found most useful.

The statistics show that the tampon in every case at once stopped the hemorrhage, and that in only one case did fatal sepsis supervene, and in that case it was quite avoidable. As has been said, death resulted only when tamponade was resorted to after everything else had failed.—*Medical Chronicle.*

---

### The Action of Olive Oil on the Secretion of Bile.

Although there is nothing very new in the administration of olive oil for the relief of biliary colic, the following abstract is inserted in the hope of shedding a little light on the obscure question of how it acts to relieve the pain and other symptoms of this distressing affection.

In an article which Dr. Seigfried Rosenberg read at a meeting of the Medical Society of Berlin, entitled "The Use of Olive Oil in the Treatment of Biliary Colic," the writer reports three cases successfully treated by this method. Including his own, he finds recorded in the medical journals twenty-one cases in which the oil treatment has been tried; in nineteen of these the patients were described as much relieved or well at the time of their discharge, while in two cases only no improvement resulted.

In considering the *modus operandi* of this remedy, he takes exception to the theory which assumes that the oil in some manner finds its way into the biliary passages, where it produces a softening of the gall-stones, thus facilitating their passage along the duct. He believes that the exhibition of a large amount of oil or fat in the stomach and duodenum excites a correspondingly large flow of bile, and that this flushing of the channels with bile dislodges the calculi and cures the colic. He argues that since bile plays so important a *rôle* in the absorption of fat, it is fair to assume that the presence of fatty food in some way calls forth the bile which is so necessary for its assimilation. In his endeavor to prove this he tried a number of experiments on two dogs with permanent biliary fistulæ, giving them in the morning a large dose of oil (sometimes a quarter of a pound of solid fat of ham or bacon was substituted by way of variety), and noting each hour the amount of bile which had flowed from the fistula. In every case he found a very considerable increase in the amount and duration of the flow over that produced by an ordinary meal of carbohydrates and albuminoids. It was also considerably more than the secretion he could produce by giving salicylate of soda or bile itself, both of which he had previously regarded as active excitors of the biliary function. He concludes from his experiments that oil acts as a powerful cholagogue, perhaps the most active of any, and to this action he attributes his success in the treatment of gall-stones.

Dr. Rosenberg considers it very important to flavor and disguise the oil as much as possible, for the very idea of drinking a glass of it is extremely disgusting to patients. To remove the nauseous taste, he adds one-quarter of one per cent. of menthol and ten or fifteen per cent. of brandy ; and also advises adding the yolks of two eggs, finely divided and worked in, so as to be perfectly smooth ; this materially alters the appearance of the oil. The dose should be from five and a quarter to seven ounces (150 to 200 grammes); it is best to give about an ounce at a time, in such a way that the whole amount will be consumed in three hours.—*Therapeut. Monatshefte*, Dec., 1889. *Jour. Amer. Med. Sciences*, May, 1890.

---

WOLFF. "The covering over (ueberdecken) of defects of skin, bone, and widely exposed joints."—*Berlin. klin. Woch.*, No. 6, Feb. 10, 1890.

IN this paper, given as an address before the Berlin Medical Society, Wolff describes his method of treating obstinate ulcerating surfaces. The method is not a new one, but the extent to which our author uses it is new, remarkably successful, and likely to be widely used.

A short abstract of one of his cases will best explain his treatment. A young man of twenty had, four and a half years ago, primary infectious osteomyelitis of the right tibia. This left, under the tuberosity of the tibia, a fistula leading into the medullary cavity, and the discharge from it had occasioned a very troublesome eczema of all the leg beneath it. Wolff laid open the medullary cavity, and with chisel and spoon removed all the diseased bone. He next cut away all the unhealthy skin round the fistulous opening, and then detached the healthy skin from the underlying tissues for about 4 to 5 cm. at each side and 8 cm. above and below the space to be covered. The margins of the wound were then refreshed and the loosened skin sutured over it. The skin had not been sufficiently detached at the sides, and the result was tension, sloughing, and a worse condition of things than at first; and, finally, there came to be a hole in the bone large enough to hold a walnut, and, besides, a space devoid of skin 6 cm. broad and 8 cm. long (2 in. broad and about 3 in. long). To meet this state of affairs, Wolff detached the skin right up to the patella above, and below down to the middle of the leg, whilst behind he left it adherent to the calf for about 1½ cm., his fingers as he separated the skin with them almost meeting each other in the middle line. Every now and then during the process he tried if he had skin enough to cover the opening, and stopped when he found he had sufficient. In detaching he mainly used his fingers, only requiring a scissors or probe-pointed bistoury to sever the more obstinate bands of connective tissue. The loosened skin-flaps were then drawn over the wound and sewed in a straight line with a running suture (the stitches on each side 5 cm. from the edge), so that the borders of the flaps slightly overlapped each other. Next, a row of small stitches brought the margins of the skin into close careful apposition. No drainage tube was employed; only a bit of skin as large as a pea cut out of the centre of the suture served the same purpose. Small wedgelets of iodoform gauze, introduced under the large stitches, prevented them cutting their way through the skin. In nine days, with the exception of the little opening made for drainage, the wound had healed by the first intention. In thirty-three days the whole wound was healed, without even a fistula.

In another case, a large exposed surface, reaching from beyond the condyle of the femur above till beneath the tuberosity of the tibia below, was treated in a similar bold manner, with like success. In twelve days the whole wound had healed by the first intention.

Of course, he says, in conclusion, the method has its limits, and will fail when the stretching power of the skin is over-estimated or when it lacks vitality enough. In ulcers of the lower part of the leg he has been very much disappointed, as also in inguinal buboes.—*Medical Chronicle.*

---

### THE THERAPEUTIC USE OF CAMPHORIC ACID.

Since the publication of articles on this subject by Fürbringer and by Reichert, camphoric acid has been extensively introduced in the clinic of Professor Mosler, at the Royal University Hospital at Griefswald, and a report of the results obtained therewith forms the subject-matter of an interesting communication by DR. BERNHARDT HARTLEIB in a recent number of the *Wiener Medi-*

*cinische Press* (Feb. 23, 1890). The action of this drug was studied in three classes of diseases; namely, in acute and chronic catarrhal affections of the respiratory mucous membrane, in acute and chronic cystitis, and in the night-sweats of phthisis.

In the first class of cases, ordinary sore throat (angina) and catarrhal pharyngitis were much improved by using a one-half to one per cent. solution as a gargle. The patients noticed their own rapid improvement, and expressed their satisfaction with the treatment, yet it is doubtful if it really acted more promptly than other remedies. Fourteen cases of laryngitis, in which the solution was either sprayed on or applied with a brush, also furnished gratifying results. But the inhalations of the drug administered for chronic bronchitis or pulmonary tuberculosis were not as successful. Eighteen patients received this form of treatment, and no improvement in their physical condition could be claimed, although several declared that they were relieved of the feeling of weight in the chest, so that they breathed more easily. The strength of the solution used for inhalation varied from one to four per cent. As camphoric acid is only solube in water to the extent of nine-tenths of one per cent., alcohol, glycerine, and water made alkaline by the addition of bicarbonate of soda, were separately used as media to dissolve it.

Excellent results were obtained in the second class of cases, *i. e.*, cystitis. There were ten of these cases under treatment with camphoric acid, five acute and five chronic. Of the latter, three were reported well six weeks after all medication had been stopped, the fourth was unfortunately obliged to leave before it was prudent to do so, and the fifth, a very obstinate case of four years' duration, was at first much improved, but the treatment was changed, and ten days later he had a relapse. Of the five acute cases, four made a complete recovery before leaving the hospital, while the fifth, still having a slight trace of pus in the urine, left against advice.

In practice, the writer recommends washing out the bladder twice a day with a one-half of one per cent. solution, and leaving an ounce or two inside when the catheter is withdrawn. After the camphoric acid has been employed in this way for several weeks, the strength of the solution may be increased. Where pyelitis is present, as well as cystitis, the drug may be simultaneously administered by the mouth. If the bladder be very irritable, local treatment should be suspended and eight grains (one-half of a gramme) given by the mouth three times a day. For convenience in irrigating the bladder, Hartleib advises keeping a twenty per cent. solution of camphoric acid in alcohol; three drachms of this in a pint of lukewarm water furnish approximately the required one-half per cent. solution. Among the cases of cystitis was one of gonorrhœal origin; it was apparently cured.

In treating the third class of cases, the night-sweats of phthisis, the usual amount given was fifteen grains (one gramme) at bed time, although in obstinate cases thirty grains (two grammes) may be required. Hartleib does not believe it necessary to give as much as Fürbringer had recommended. Thirteen patients of this class received treatment, twelve of them with the result of stopping the night-sweats; the thirteenth, a phthisical patient in the surgical wards, with extensive tubercular disease of bone, received the usual amount (fifteen grains) for several nights without producing any effect; a larger dose was not tried.

The disagreeable after-effects of camphoric acid were few. One patient, who had taken fifteen grains (one gramme) three times a day, complained of severe pain in the region of the kidneys, which disappeared on stopping all treatment. Two patients, whose bladders were frequently irrigated, developed a slight swelling of the glans, which soon subsided. One consumptive patient vomited a dose of thirty grains (two grammes) of the acid, but retained half the amount without difficulty. Continued use of it does not appear to affect the stomach or intestine, in fact its action in proper doses is quite harmless. *Jour. Amer. Med. Sciences*, May, 1890.

---

### CHLORALAMIDE AS A HYPNOTIC FOR THE INSANE.

"Of all the somnifacients discovered of late years," says Dr. S. A. K. Strahan, in *The Lancet* (February 15, 1890), "none is likely to prove a more certain sleep-producer, and at the same time,be more innocent or otherwise agreeable, than chloralamide." Among the insane it has acted to induce sleep as certainly, if not as promptly, as chloral itself. It rarely fails to produce the desired effect, even in extreme maniacal excitement, and when it did fail it did not increase that excitement as other drugs have been found to do; on the contrary, it always calmed the patient more or less.

From thirty-five to fifty grains were usually sufficient to give from five to ten or more hours' refreshing sleep, and with fifty-five grains some effect was always obtained, even in the worst case of subacute mania or excitement following epileptic seizures. Patients were calm, and yet did not sleep heavily—that is to say, it was always easy to rouse them—and they usually declared themselves much refreshed on waking. In most cases sleep came on about an hour and a half after taking the medicine, although this interval varied from twenty minutes to three hours; two patients, however, were a little drowsy on the following day, and several, contrary to their custom, slept the next night without any medicine.

Chloralamide was not observed to affect the temperature, respiration, or pulse; in fact, the writer believes with Kuy, of Strassburg, that it acts precisely like chloral, only without any depressant effect on the heart. Headache, and the ataxic symptoms, sometimes seen after the use of sulphonal, were not noted; nor was any gastric trouble or loss of appetite attributed to its use. In fact, it is a very useful and safe hypnotic, equal but not superior in efficiency to paraldehyde; it is, however, pleasanter to take, and is free from the disadvantage of imparting any disagreeable odor to the breath.—*Jour. Amer. Med. Sciences, May, 1890.*

---

In a paper on "The Use and Abuse of Soap and Water," Dr. B. Merrill Ricketts concludes as follows:

To overcome the disadvantages enumerated in the foregoing paper, I have succeeded in obtaining, with the aid of Professor Karl Langenbeck, what might be called *saponaceous cream*, the two things most important being—

1. The attainment of an absolutely neutral soap containing neither an excess of acid or alkali, especially the latter.

2. One composed of fat or oil not prone to decomposition either before or after saponification.

It has been determined that olive-oil is the best to attain such an end. While cocoanut-oil and palm-oil gives more lather, they contain properties conducive to rancidity.

After chemically testing olive-oil and soda, water is added, the result being a saponification into a smooth, gelatinous, opaque, odorless and neutral mass, which gives but little lather when used with water.

To this is added the fresh albumen of an egg thoroughly incorporated by means of an ordinary egg-beater. A few drops of oil of roses, bergamot or bitter almonds being added will give it an agreeable odor.

With this may be incorporated many medical agents varying in strength as follows: Ichthyol, 5 per cent.; iodol, 1 per cent.; boric acid, 5 per cent.; thymol, 3 per cent.; sulphur, 5 per cent.; eucalyptol, 5 per cent.; hydrargyri bichloridi, ½ per cent.; tar, 10 per cent.; carbolic acid, 5 per cent. In conclusion, I will say that after one year's experience I can report favorably upon its use.

---

### SUPPOSITORIES AGAINST DYSMENORRHŒA.

℞. Extr, fl. cannab. indicæ., gr. ¼.

Extr. fl. belladonnæ, gr. ¼.

Butyr. cacao, ℥j ss. M.

This is sufficient for one suppository; five such ones may be made. One suppository may be introduced every evening, commencing the fifth day before the menses.—*Journ. de Médicine de Paris, February 9th, 1890. Annals of Gynæcology aud Pædiatry.*

---

### ENDOMETRITIS.

For subacute cases of this disease Terrier uses medicated pencils introduced into the uterus. He recommends :

℞. Iodoformi, gr. cl.
Gummi tragacanth, gr. vijss.
Glycerini,
Aquæ destill., *aa* q.s.
Ut. fiant bacilla (pencils) No. 10.

The pencils made according to this formula are said to be about the size of a stick of nitrate of silver. Resorcin or salol may be used instead of iodoform.—*Canada Lancet*, December, 1889.

### BUTYL CHLORAL IN NEURALGIA OF PREGNANCY.

Butyl chloral has been found to have, as well as a diaphoretic, a specific action upon the trigeminus nerve, which may be anæsthetized by 1-3 grain, internally. Professor Leibreich, of Berlin, prescribes it as follows:

℞. Butyl chloral, gr. xl.-lxxv.

Alcohol rect., fl ʒ ijss.

Glycerini, fl ʒv.

Aq. dest., fl ℥iv.

S. Two to four spoonfuls at once.— *Wiener. klin. Wochenschr.*, 48, 1889.

---

### AN INJECTION AGAINST LEUCORRHŒA AND BLENNORRHŒA IN WOMEN.

(Lutaud.)

℞. Creolin, gtt. xxx.
Ext. fluid hydr. canad., fl ʒijss.

S. Two teaspoonfuls in a pint of warm water to be used at one injection.

As a urethral injection the following formula is used:

℞ Extr. fluid hydrast. canad. gtt. xxx.
Creolin, gtt. x.
Aquæ, fl. ℥viii.

S. Use pure as a urethral injection.—*Journ. de Médicine de Paris*, 1889, No. 48.

